# Exophagy of annexin A2 via RAB11, RAB8A and RAB27A in IFN-γ-stimulated lung epithelial cells

**DOI:** 10.1038/s41598-017-06076-4

**Published:** 2017-07-18

**Authors:** Ying-Da Chen, Yi-Ting Fang, Yi-Lin Cheng, Chiou-Feng Lin, Li-Jin Hsu, Shu-Ying Wang, Robert Anderson, Chih-Peng Chang, Yee-Shin Lin

**Affiliations:** 1 Institute of Basic Medical Sciences, College of Medicine, National Cheng Kung University, Tainan, Taiwan; 2 Department of Microbiology and Immunology, College of Medicine, National Cheng Kung University, Tainan, Taiwan; 3 Center of Infectious Disease and Signaling Research, College of Medicine, National Cheng Kung University, Tainan, Taiwan; 4 Department of Microbiology and Immunology, College of Medicine, Taipei Medical University, Taipei, Taiwan; 5 Department of Medical Laboratory Science and Biotechnology, College of Medicine, National Cheng Kung University, Tainan, Taiwan; 6 Departments of Microbiology & Immunology and Pediatrics, and Canadian Center for Vaccinology, Dalhousie University, Halifax, Canada

## Abstract

Annexin A2 (ANXA2), a phospholipid-binding protein, has multiple biological functions depending on its cellular localization. We previously demonstrated that IFN-γ-triggered ANXA2 secretion is associated with exosomal release. Here, we show that IFN-γ-induced autophagy is essential for the extracellular secretion of ANXA2 in lung epithelial cells. We observed colocalization of ANXA2-containing autophagosomes with multivesicular bodies (MVBs) after IFN-γ stimulation, followed by exosomal release. IFN-γ-induced exophagic release of ANXA2 could not be observed in *ATG5*-silenced or mutant *RAB11*-expressing cells. Furthermore, knockdown of *RAB8A* and *RAB27A*, but not *RAB27B*, reduced IFN-γ-triggered ANXA2 secretion. Surface translocation of ANXA2 enhanced efferocytosis by epithelial cells, and inhibition of different exophagic steps, including autophagosome formation, fusion of autophagosomes with MVBs, and fusion of amphisomes with plasma membrane, reduced ANXA2-mediated efferocytosis. Our data reveal a novel route of IFN-γ-induced exophagy of ANXA2.

## Introduction

Annexin A2 (ANXA2), a Ca^2+^-dependent membrane-binding protein, can distribute to the nucleus, the cytosolic membrane of organelles, as well as the inner and outer leaflets of the plasma membrane in different cell types, including macrophages, endothelium, epithelium, and tumor cells^1–3^. ANXA2 has broad impact in fundamental biological processes including cell proliferation, endocytosis, exocytosis, cytoskeletal rearrangement, intracellular signal transduction, fibrinolysis response, and cell migration^4–10^. Importantly, extracellular ANXA2 is associated with many human diseases, such as cancers, inflammatory disorders and autoimmune diseases^11–13^. It has been previously reported that ANXA2, which lacks any signal peptide for targeting to the endoplasmic reticulum (ER), is transported onto the cell surface^14^. Proteins lacking signal peptides such as IL-1 have been found to be released from cells in exosomal-like vesicles via an unconventional secretion pathway^15^. We previously demonstrated that ANXA2 is transported to the extracellular surface via exosomal release after IFN-γ stimulation^16^. However, the molecular mechanism of ANXA2 trafficking from cytoplasm to extracellular matrix is not known.

Autophagy is an important process which regulates key biological functions, such as inflammation and aging, as well as human diseases, including cancers, infections and neurodegenerative disorders^17–22^. Although the central dogma of autophagy is the degradation of long-lived proteins and cytoplasmic organelles in eukaryotic cells, it has recently been demonstrated to participate in the unconventional secretion pathway of proteins such as Acb1 proteins of yeast^23, 24^, and IL-1β and HMGB1 of mammalian cells^25, 26^. These autophagic secretion processes of cytosolic proteins lacking signal peptides are known as exophagy^27^.

Autophagy is closely related with functional multivesicular bodies (MVBs), the late endosomes generated during cell endocytosis^28^. In mammalian cells, mature autophagosomes fuse with MVBs to generate the amphisomes, which subsequently fuse with lysosomes to form autolysosomes which finally degrade incorporated materials^29^. It has been shown that RAB proteins, members of the RAS GTPase superfamily, play an important role in exosome secretion and autophagy^30, 31^. RAB27A and RAB27B regulate different steps during exosome secretion^32^. RAB11 has been proved to regulate the fusion of autophagosomes with MVBs^33^ whereas RAB8A plays a critical role in exophagy^34^. We previously demonstrated that IFN-γ-induced epithelial cell surface expression of ANXA2 occurs through an unconventional secretion pathway of exosomal release^16^. In the present study, we further investigated the potential roles of autophagic pathway in exosomal secretion of ANXA2 from cytoplasm to extracellular matrix after IFN-γ stimulation. Moreover, autophagy has been demonstrated to enhance phagocytosis of apoptotic cells, called efferocytosis^35^. We previously showed that IFN-γ-induced surface expression of the ANXA2/S100A10 complex enhances efferocytosis by lung epithelial cells^16^. In this study, we provide a mechanistic insight into the ANXA2-mediated efferocytosis via a RAB11, RAB8A and RAB27A-controlled exophagic pathway.

## Results

### IFN-γ-induced autophagy is essential for extracellular secretion of ANXA2 in lung epithelial cells

**Figure 1:**
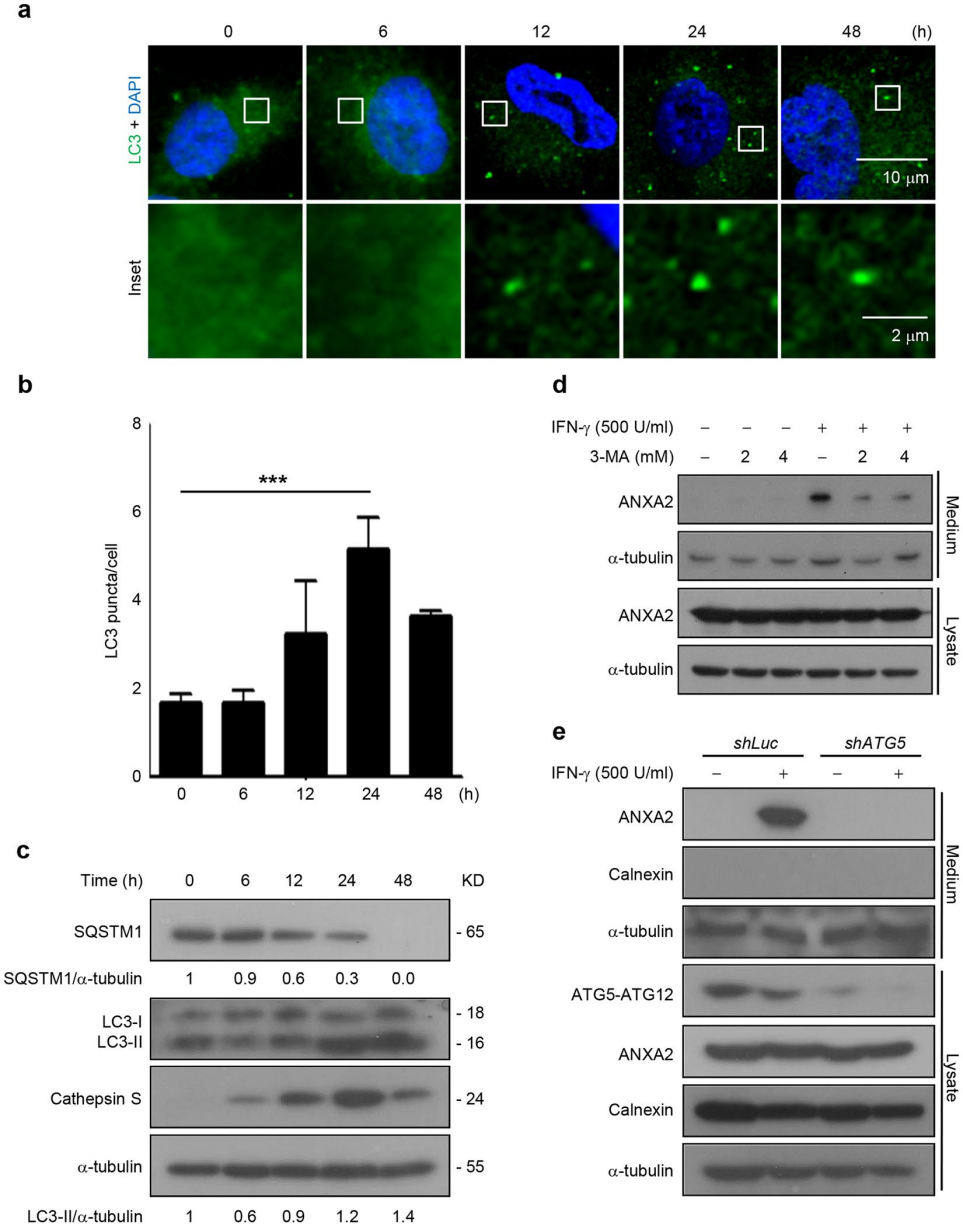
IFN-γ-induced autophagy is required for extracellular secretion of ANXA2 in human lung epithelial cells. (**a**) A549 cells were treated with or without 500 U/ml IFN-γ for different time periods. After fixation and permeabilization, cells were stained with anti-LC3 (green) and DAPI (blue) to detect LC3 puncta and nuclei, respectively, and observed by confocal microscopy. Scale bar: 10 μm (2 μm in insets). (**b**) The quantification of LC3 puncta per cell is shown. Data are represented as mean ± SD. ***P < 0.001. At least 30 cells were counted in each treatment group. (**c**) Cells were cultured with 500 U/ml IFN-γ for the indicated times. Cells were harvested and lysed after stimulation. The expression of SQSTM1/p62, LC3, cathepsin S and α-tubulin were analyzed by western blotting. The densitometric ratio of LC3-II and α-tubulin is shown. kD, molecular weight as kDa. (**d**) Cells were pretreated in the presence or absence of 3-MA for 1 h and then stimulated with or without 500 U/ml IFN-γ. After 48 h, ANXA2 and α-tubulin from cultured supernatant and lysate were analyzed by western blotting. (**e**) Cells were transfected with shRNA specifically targeting *luciferase* or *ATG5*. Cells were cultured with or without 500 U/ml IFN-γ for 48 h. ANXA2, calnexin and α-tubulin from cultured supernatant and cell lysate were analyzed by western blotting. *ATG5* knockdown efficiency was detected by western blotting.

Our previous study demonstrated that IFN-γ-induced secretion of ANXA2 is associated with an unconventional exosome secretion pathway^16^. The detailed machinery for ANXA2 trafficking from cytosol to the MVB/exosome secretion route is still unknown. First, we confirmed that IFN-γ stimulation induced autophagosome formation in human lung epithelial cells. IFN-γ stimulation induced LC3 punctation, which was most prominent after IFN-γ treatment for 24 h (Fig. 1a,b). We also observed SQSTM1/p62 degradation and LC3-I/II conversion after IFN-γ stimulation, indicating that IFN-γ induces a functional autophagic flux in lung epithelial cells (Fig. 1c). The expression of cathepsin S, a downstream signaling factor of IFN-γ^36^, was also apparent after IFN-γ treatment with a maximal level at 24 h (Fig. 1c). Our previous study showed that secretion of ANXA2 was increased after IFN-γ stimulation^16^. We investigated whether IFN-γ-induced autophagy in A549 cells is involved in the extracellular secretion of ANXA2 by treating cells with 3-MA or using *ATG5*-silenced cells to inhibit autophagy. The appearance of α-tubulin released into the supernatant from cells by the alternative secretion pathway^37^ served as control (Fig. 1d,e). The release of calnexin, an ER marker, was not detected in the culture medium (Fig. 1e), confirming that ANXA2 release was not caused by cell lysis. IFN-γ-triggered ANXA2 secretion was inhibited in 3-MA-treated cells (Fig. 1d) and *ATG5*-silenced cells (Fig. 1e). These results indicate that IFN-γ-induced autophagy is associated with extracellular secretion of ANXA2.

### IFN-γ-induced autophagy regulates exosomal secretion of ANXA2

**Figure 2:**
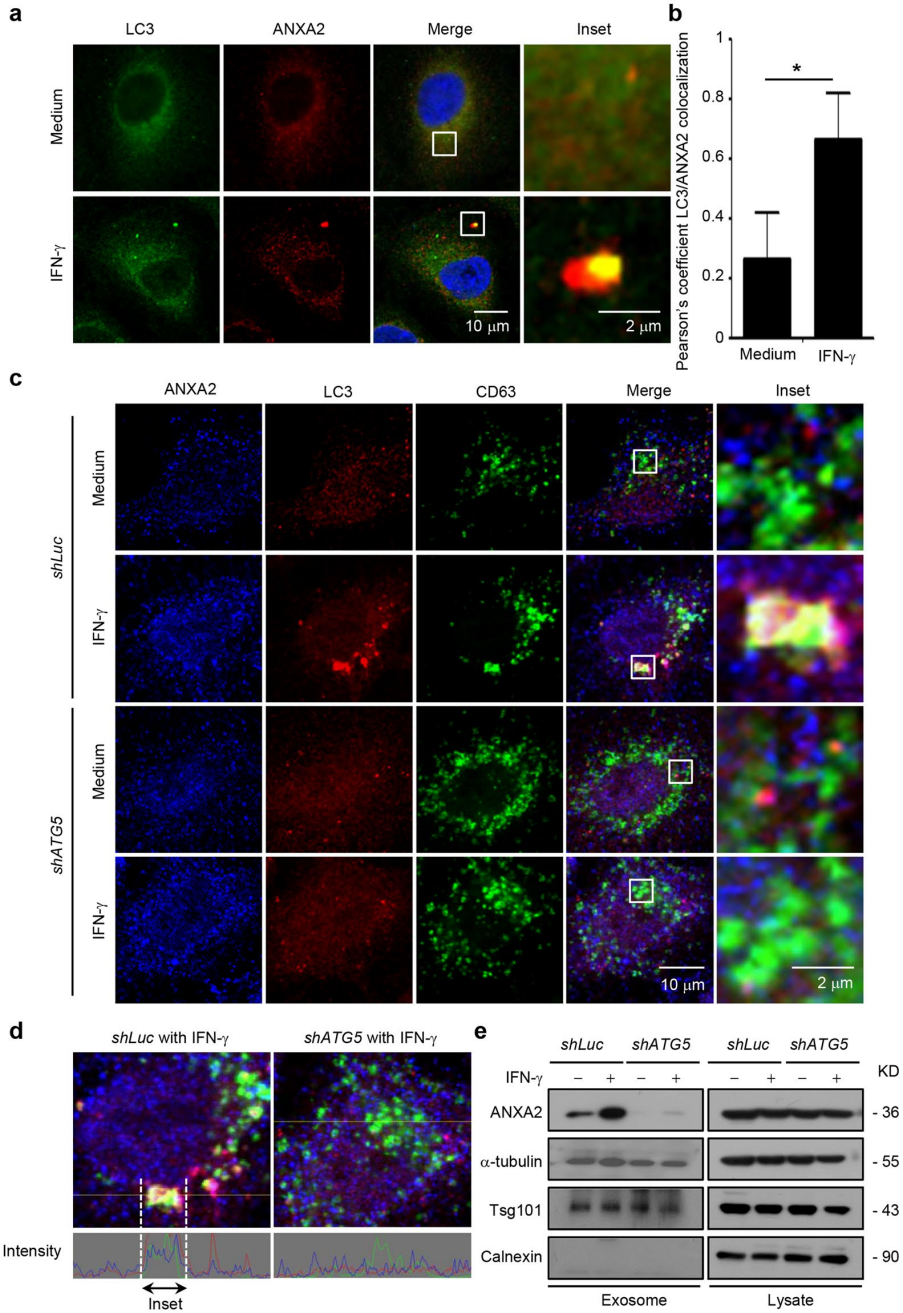
IFN-γ-triggered amphisome formation is required for ANXA2 exosomal secretion. (**a**) A549 cells were treated with or without 500 U/ml IFN-γ for 24 h. Cells were then fixed, permeabilized, and stained for LC3 (green) and ANXA2 (red). The colocalization of ANXA2 and LC3 was observed by confocal microscopy. Scale bar: 10 μm (2 μm in insets). (**b**) Quantification of colocalization is shown. Data are represented as mean ± SD. *P < 0.05. At least 3 cells were quantified in each treatment group. (**c**) Cells with stable *ATG5* knockdown and control knockdown were treated with or without 500 U/ml IFN-γ for 24 h. Cells were then fixed, permeabilized, and stained for ANXA2 (blue), LC3 (red) and CD63 (green). The colocalization of ANXA2, LC3 and CD63 was observed by confocal microscopy. Scale bar: 10 μm (2 μm in insets). (**d**) Line tracing analysis of fluorescence signal from image of *ATG5* knockdown and control cells after IFN-γ stimulation is shown. (**e**) Control and *ATG5*-silenced cells were treated with or without 500 U/ml IFN-γ for 48 h. The exosome pellets were collected. ANXA2, α-tubulin, Tsg101 (exosome marker) and calnexin from exosome pellets and total cell lysates were detected by western blotting. kD, molecular weight as kDa.

We then examined whether ANXA2 colocalized with autophagosomes and/or amphisomes after IFN-γ stimulation. The colocalization of ANXA2 with LC3 puncta was observed after IFN-γ stimulation for 24 h (Fig. 2a,b), suggesting that ANXA2 was incorporated into autophagosomes after IFN-γ stimulation. Since autophagosomes can fuse with MVBs as amphisomes^38^, this could be an important route for exosomal secretion of ANXA2. To elucidate whether ANXA2-containing autophagosomes fuse with MVBs to form amphisomes and cause the release of ANXA2, we performed confocal microscopy which showed colocalization of LC3 puncta with ANXA2 and CD63, a marker of MVBs, after IFN-γ treatment; however, no colocalization was detected in cells with autophagy deficiency (Fig. 2c,d and Supplementary Fig. 1). To confirm the IFN-γ-induced exosomal secretion of ANXA2, we collected the exosomes from the medium of A549 cells after serial centrifugation (Supplementary Fig. 2a) and detected the presence of ANXA2 on the exosomal surface (Supplementary Fig. 2b). CD9 expression on the exosomal surface was used as a marker. We also conducted sucrose gradient to isolate the exosomes (Supplementary Fig. 2c) and further confirmed that IFN-γ enhanced exosomal secretion of ANXA2 (Supplementary Fig. 2d). Furthermore, we found that IFN-γ failed to increase exosomal secretion of ANXA2 in *ATG5*-silenced cells (Fig. 2e). The results indicate that ANXA2 is released via a route of fusion of autophagosomes with MVBs, followed by fusion with the plasma membrane for exosomal release.

### RAB11 is required for exophagy of ANXA2

**Figure 3:**
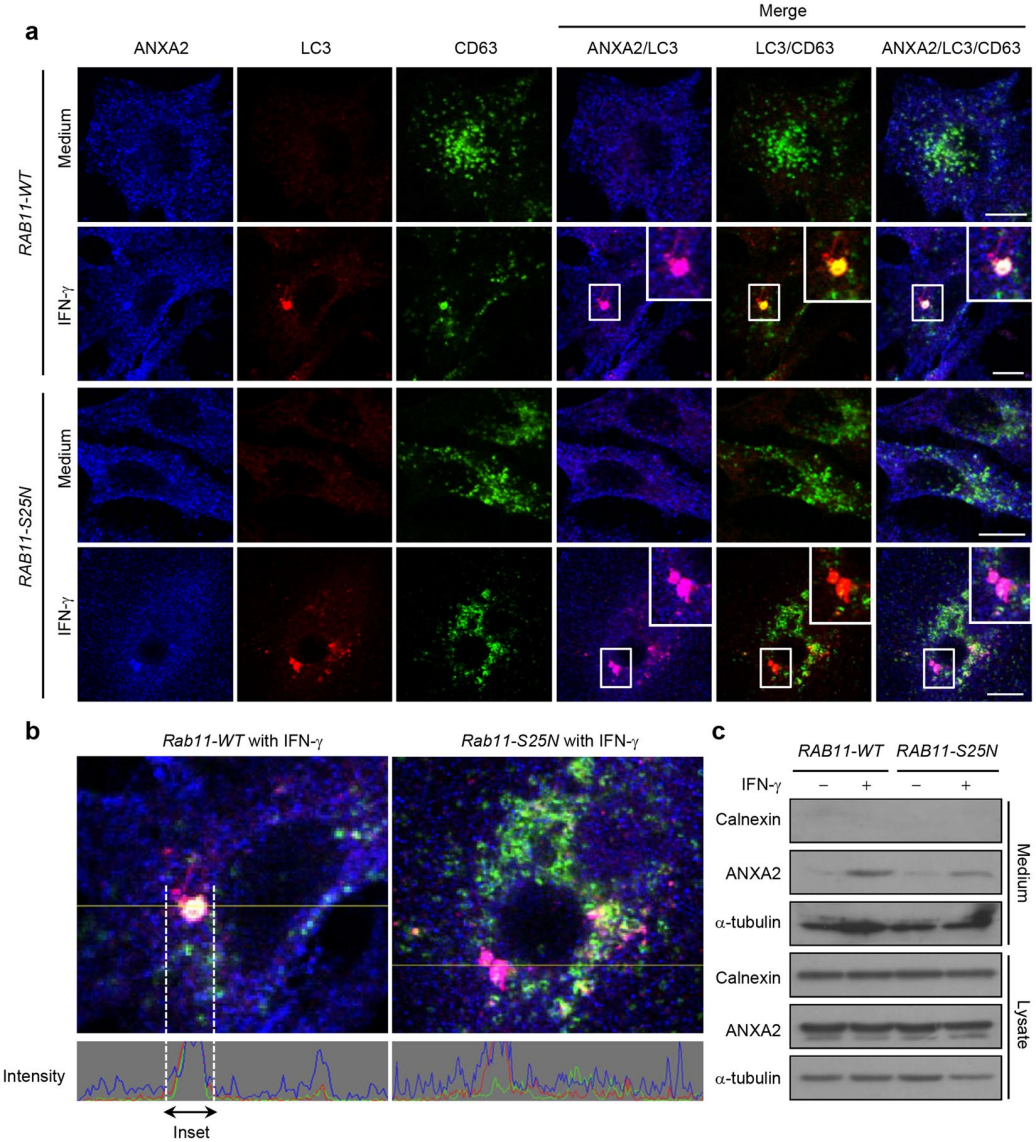
Blocking the fusion of autophagosomes with MVBs decreases ANXA2 colocalization with amphisome and exosomal secretion of ANXA2. (**a**) A549 cells were transfected with a *pcDNA*-*RAB11wt* or *pcDNA*-*RAB11S25N* plasmid and incubated with or without 500 U/ml IFN-γ for 24 h. Cells were then fixed, permeabilized and stained for ANXA2 (blue), LC3 (red) and CD63 (green). The colocalization of ANXA2, LC3 and CD63 was observed by confocal microscopy. Scale bar: 10 μm. (**b**) Line tracing analysis of fluorescence signal from image in (**a**) of *pcDNA*-*RAB11wt*- and *pcDNA*-*RAB11S25N*-expressing cells after IFN-γ stimulation is shown. (**c**) *pcDNA*-*RAB11wt*- and *pcDNA*-*RAB11S25N*-expressing cells were treated with or without 500 U/ml IFN-γ for 48 h. The media from *pcDNA*-*RAB11wt*- and *pcDNA*-*RAB11S25N*-expressing cells were collected. ANXA2, α-tubulin and calnexin from the media and total cell lysates were detected by western blotting.

Fusion between autophagosomes and MVBs is a critical event which allows the translocation of ANXA2 from autophagosomes to MVBs. A previous study showed that RAB11 is required for fusion of MVBs with autophagosomes^33^. We therefore transfected A549 cells with a plasmid expressing wild-type (WT) RAB11 or a dominant interfering RAB11S25N mutant protein, and subsequently observed autophagosomes and MVBs, by LC3 puncta and CD63 staining, respectively. IFN-γ stimulation triggered ANXA2 colocalization with LC3 puncta similarly in *RAB11WT*- and *RAB11S25N*-expressing cells (Fig. 3a; ANXA2 and LC3 merged cells). However, the colocalization of CD63 and LC3 puncta was decreased after IFN-γ stimulation in *RAB11S25N*-expressing cells as compared with WT cells (Fig. 3a; CD63 and LC3 merged cells). Overexpression of *RAB11S25N* mutant inhibited the amphisome formation from the fusion of autophagosomes with MVBs. Furthermore, colocalization of ANXA2 with amphisomes (CD63 and LC3 merged cells) was also inhibited in *RAB11S25N*-expressing cells (Fig. 3a,b; ANXA2, CD63 and LC3 merged cells). These results indicate that RAB11 is essential for transporting ANXA2 to the amphisome. To elucidate whether RAB11-mediated fusion of autophagosomes and MVBs is an important route for ANXA2 secretion, we collected the medium from the WT or *RAB11S25N*-expressing cells and observed that IFN-γ-induced ANXA2 release was decreased in *RAB11S25N*-expressing cells as compared with WT cells (Fig. 3c).

### Amphisome/lysosome fusion is not required for ANXA2 exosomal secretion

**Figure 4:**
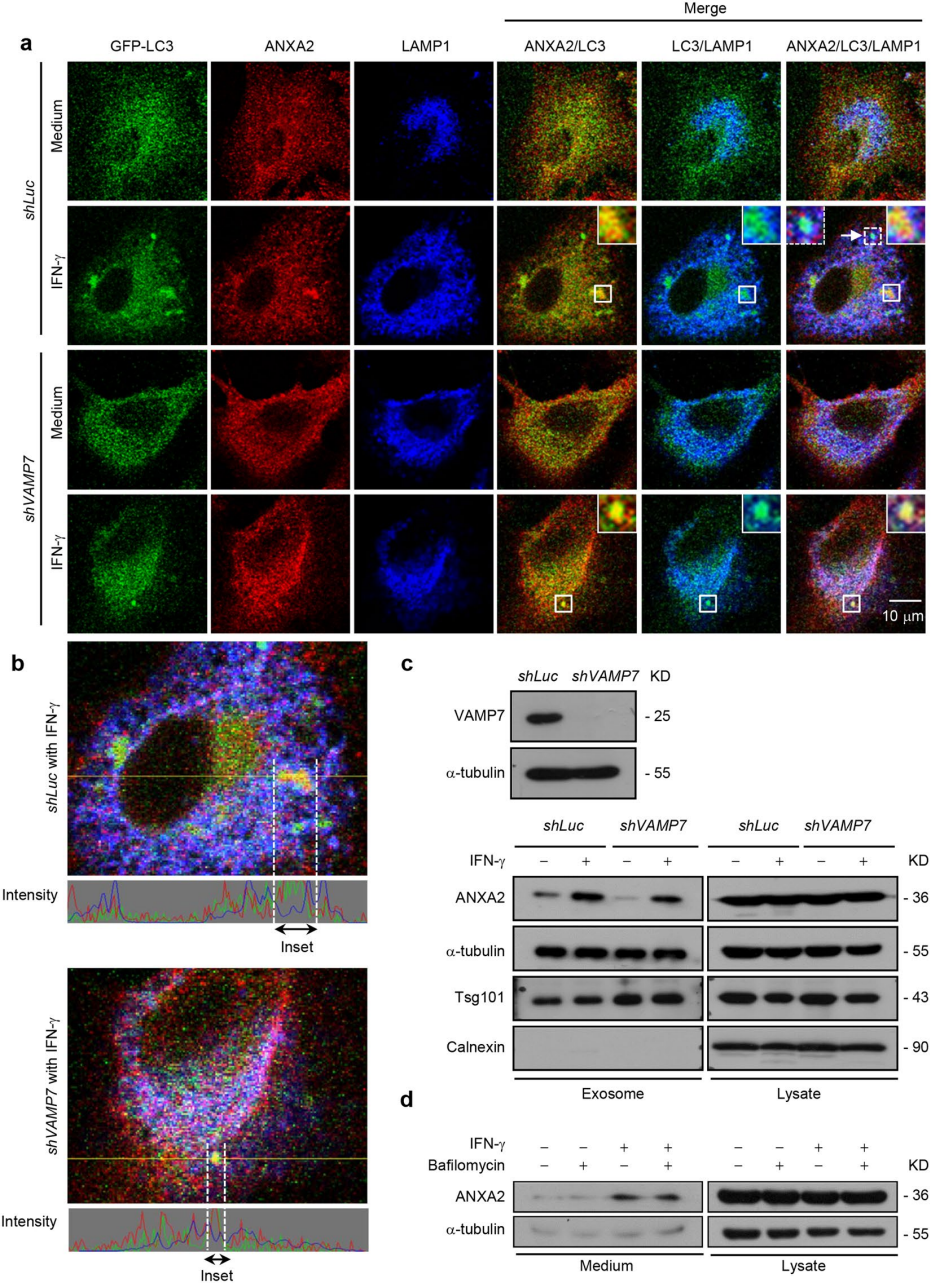
Amphisome/lysosome fusion is not required for autophagy-mediated exosomal secretion of ANXA2. (**a**) Cells with *VAMP7* knockdown and control knockdown were transfected with a *GFP*-*LC3* plasmid and treated with or without 500 U/ml IFN-γ for 24 h. Cells were then fixed, permeabilized, and stained for ANXA2 (red) and LAMP1 (blue). The colocalization of ANXA2, GFP-LC3 and LAMP1 was observed by confocal microscopy. Scale bar: 10 μm. The arrow and dotted inset mark an autolysosome. (**b**) Line tracing analysis of fluorescence signal from image in (**a**) of *VAMP7* knockdown and control knockdown cells after IFN-γ stimulation is shown. (**c**) *VAMP7* knockdown efficiency was detected by western blotting. Control and *VAMP7*-silenced cells were treated with or without 500 U/ml IFN-γ for 48 h. The exosome pellets were collected. ANXA2, α-tubulin, Tsg101 and calnexin from exosome pellets and total cell lysates were detected by western blotting. kD, molecular weight as kDa. (**d**) A549 cells were incubated with 500 U/ml IFN-γ in the presence or absence of 5 nM bafilomycin A1 for 48 h. ANXA2 and α-tubulin from cultured supernatant and total cell lysate were analyzed by western blotting. kD, molecular weight as kDa.

In autophagic flux, the autophagosome and amphisome fuse with the lysosome to degrade cargo^29^. We therefore determined whether the lysosome is involved in ANXA2 secretion. VAMP7 is the v-SNARE protein on MVB that participates in the fusion of the MVB with the lysosome^39, 40^. *VAMP7* was knocked down to inhibit amphisome/lysosome fusion. The results showed that the formation of ANXA2-containing amphisomes was not affected by *VAMP7* knockdown (Supplementary Fig. 3). Further study showed that while IFN-γ stimulation induced ANXA2 relocation with LC3 puncta, the colocalization of ANXA2 with LAMP1, a lysosomal marker, was not observed in either control or *VAMP7* knockdown cells (Fig. 4a,b). These data indicate that ANXA2-containing amphisome does not fuse with the lysosome. Furthermore, the colocalization of LC3 with LAMP1 in control knockdown cells (Fig. 4a; white arrow and dotted inset) indicates normal autolysosome formation. In the exosomal fraction, our results showed that ANXA2-containing exosomes were found after IFN-γ stimulation both in control and *VAMP7*-silenced cells (Fig. 4c). We further inhibited autophagic flux with bafilomycin A1 and found that ANXA2 release after IFN-γ stimulation was not affected (Fig. 4d). It has been shown that RAB7 is required for the transfer of cargo from the MVBs to the lysosome^41^. We found that the colocalization of ANXA2 with LAMP1 was also not observed in *RAB7*-silenced cells (Supplementary Fig. 4a,b) and the IFN-γ-induced ANXA2 release was not affected by knockdown of *RAB7* (Supplementary Fig. 4c). These data suggest that exosomal secretion of ANXA2 does not occur through the fusion of ANXA2-containing amphisomes with lysosomes.

### RAB8A and RAB27A regulate ANXA2 secretion

**Figure 5:**
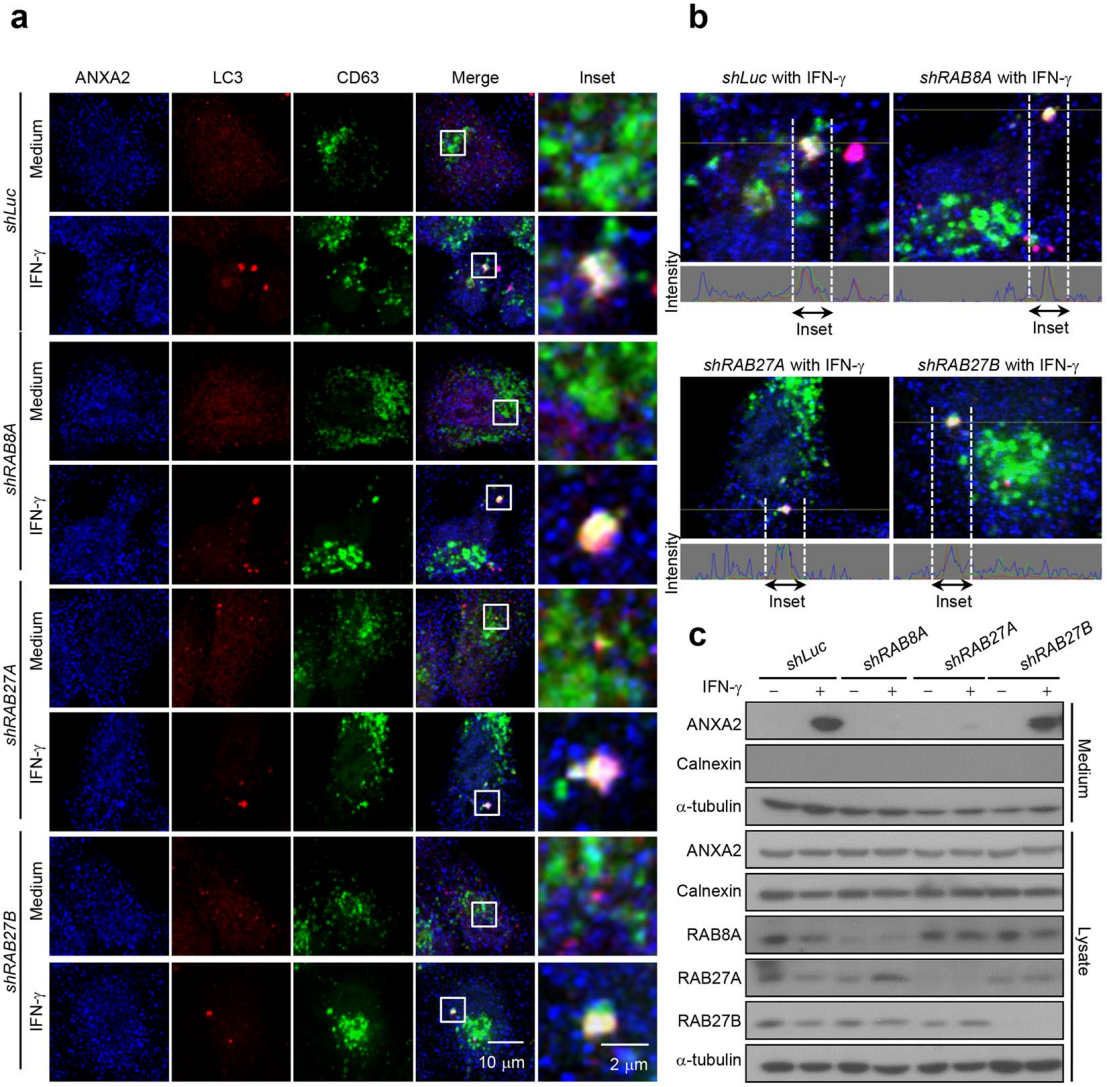
ANXA2 secretion pathway is regulated by RAB8A and RAB27A. (**a**) Cells with *RAB8A*, *RAB27A*, *RAB27B* knockdown and control knockdown were cultured with or without 500 U/ml IFN-γ for 24 h. Cells were then fixed, permeabilized and stained for ANXA2 (blue), LC3 (red) and CD63 (green). The colocalization of ANXA2, LC3 and CD63 was observed by confocal microscopy. Scale bar: 10 μm (2 μm in insets). (**b**) Line tracing analysis of fluorescence signal from image in (**a**) of *RAB8A*, *RAB27A*, *RAB27B* knockdown and control knockdown cells after IFN-γ stimulation is shown. (**c**) *RAB8A*, *RAB27A*, *RAB27B* knockdown and control knockdown cells were cultured with or without 500 U/ml IFN-γ for 48 h. ANXA2, calnexin and α-tubulin from cultured supernatant and total cell lysate were analyzed by western blotting.

Since ANXA2-containing amphisomes were detected after IFN-γ stimulation, we further investigated which proteins were responsible for the fusion of ANXA2-containing amphisomes with the plasma membrane. It has been shown that RAB8A controls secretory autophagy^34^ and RAB27A and RAB27B control the transport of MVBs to the plasma membrane^31^. Our data showed that the colocalization of ANXA2 with LC3 and CD63 can be detected in control, *RAB8A*, *RAB27A* and *RAB27B* knockdown cells after IFN-γ stimulation (Fig. 5a,b). These results suggested that the ANXA2-containing amphisome formation was not affected by knockdown of these RAB genes. However, ANXA2 release was inhibited in *RAB8A* and *RAB27A* but not *RAB27B* knockdown cells after IFN-γ stimulation (Fig. 5c). These data indicate that RAB8A and RAB27A, but not RAB27B, are involved in the fusion of ANXA2-containing amphisomes with the plasma membrane.

### Inhibition of exophagy reduces ANXA2-mediated efferocytosis

**Figure 6:**
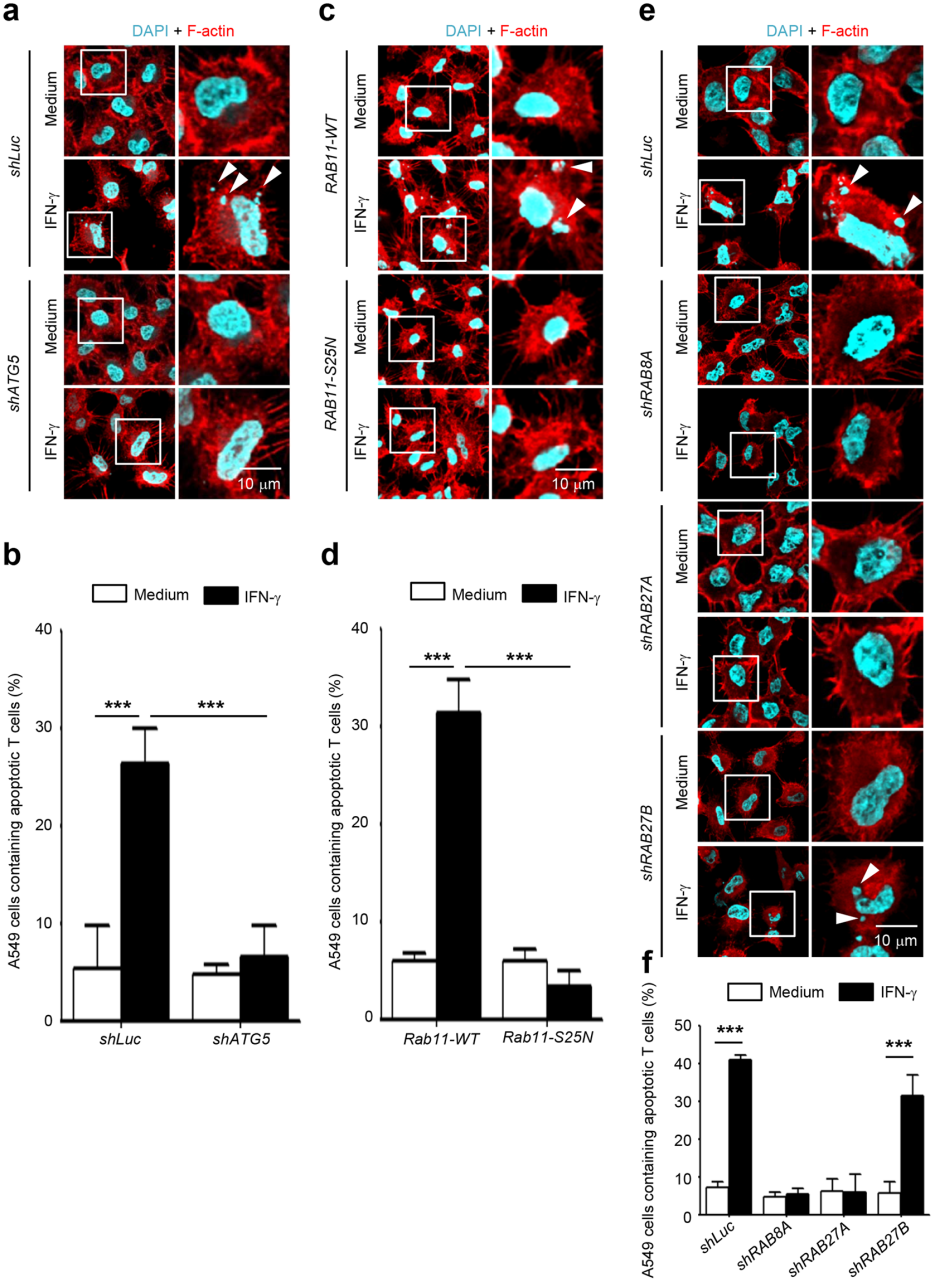
Inhibition of exophagy of ANXA2 reduces ANXA2-mediated efferocytosis. Jurkat T cells were treated with 50 μM etoposide for 24 h to induce cell apoptosis. *ATG5*, *RAB8A*, *RAB27A*, or *RAB27B* knockdown or *pcDNA*-*RAB11wt*- and *pcDNA*-*RAB11S25N*-overexpressing A549 cells were treated with 500 U/ml IFN-γ for 48 h. After treatment, these A549 cells were co-cultured with apoptotic Jurkat T cells for 2 h. Cells were then washed, fixed, permeabilized and stained with DAPI and rhodamine-phalloidin for nucleus and F-actin, respectively. Cells were mounted and analyzed by confocal microscopy. The nuclei of apoptotic T cells were indicated by white arrowheads in *ATG5* knockdown cells (**a**), *pcDNA*-*RAB11wt*- and *pcDNA*-*RAB11S25N*-overexpressing cells (**c**), and *RAB8A*, *RAB27A*, *RAB27B* knockdown cells (**e**). Scale bar: 10 μm. Cells containing nuclei fragments were quantified in *ATG5* knockdown cells (**b**), *pcDNA*-*RAB11wt*- and *pcDNA*-*RAB11S25N*-overexpressing cells (**d**), and *RAB8A*, *RAB27A*, *RAB27B* knockdown cells (**f**). Data are represented as mean ± SD. ***P < 0.001. At least 70 cells were counted in each treatment group.

We previously showed that IFN-γ-induced surface expression of ANXA2 enhances efferocytosis^16^. Since ANXA2 was secreted through exophagy, inhibition of exophagy would reduce ANXA2-mediated efferocytosis. We inhibited different steps of exophagy leading to ANXA2 secretion, including autophagosome formation, fusion of autophagosomes with MVBs, and fusion of ANXA2-containing amphisomes with the plasma membrane, and then assayed ANXA2-mediated efferocytosis. To assay efferocytosis, we used confocal microscopy to determine the condensed DAPI-stained nuclei of apoptotic cells which were ingested in A549 cell monolayers. Jurkat T cells were treated with etoposide to induce cell apoptosis and further co-cultured with A549 cells. The results showed that inhibition of autophagosome formation by *ATG5* knockdown reduced efferocytosis by A549 cells after IFN-γ stimulation (Fig. 6a,b). Similar results were observed in *RAB11S25N*-expressing cells in which fusion of autophagosomes with MVBs was inhibited (Fig. 6c,d). Efferocytosis was also reduced in *RAB8A* and *RAB27A* knockdown cells in which fusion of ANXA2-containing amphisomes with the plasma membrane was inhibited (Fig. 6e,f). Consistent with the above-mentioned results that *RAB27B* knockdown did not inhibit ANXA2 secretion (Fig. 5c), efferocytosis was not reduced in *RAB27B* knockdown cells (Fig. 6e,f). In a previous study, we demonstrated the IFN-γ-induced surface expression of ANXA2 by eluting the surface ANXA2^16^. To confirm the reduction of ANXA2 surface expression in *ATG5*, *RAB8A* and *RAB27A* knockdown cells, we eluted and detected the surface ANXA2 by western blotting (Supplementary Fig. 5). The IFN-γ-enhanced surface expression of ANXA2 was reduced in *ATG5*, *RAB8A* and *RAB27A* knockdown cells.

## Discussion

**Figure 7:**
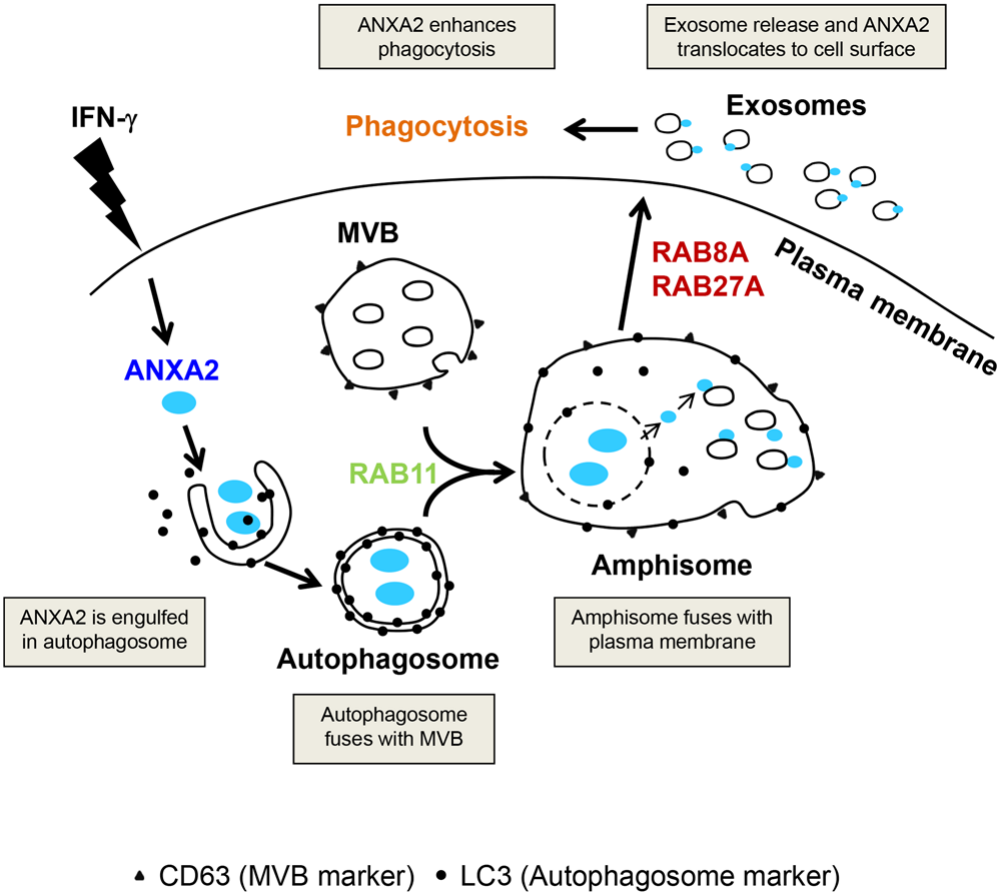
Transportation route for exophagy of ANXA2 in IFN-γ-stimulated lung epithelial cells. ANXA2 is targeted by IFN-γ-induced autophagosomes. The ANXA2-containing autophagosomes fuse with MVBs to form ANXA2-containing amphisomes. ANXA2-containing amphisomes further fuse with the plasma membrane and ANXA2 is then released. Fusion of autophagosomes with MVBs is controlled by RAB11, and fusion of amphisomes with the plasma membrane is regulated by RAB8A and RAB27A. ANXA2 translocates from cytosol to cell surface and enhances efferocytosis by human lung epithelial cells.

In this study, we reveal that exophagy is involved in ANXA2 exosomal secretion. In IFN-γ-stimulated signaling, ANXA2 is engulfed in autophagosomes. RAB11 regulates the fusion of ANXA2-containing autophagosomes with MVBs to form an ANXA2-containing amphisome. RAB8A and RAB27A control the further fusion of ANXA2-containing amphisomes with the plasma membrane for the release of ANXA2-containing exosomes. Thus, ANXA2 translocates from cytosol to cell surface and enhances efferocytosis by lung epithelial cells (Fig. 7).

Extracellular ANXA2 has been demonstrated to be involved in many cancers. ANXA2 released to the extracellular matrix increases the resistance of pancreatic cancer cells to anticancer drugs^42^. ANXA2 on the tumor cell surface mediates tumor invasion and promotes malignant progression^43^. ANXA2 also mediates adhesion of breast cancer cells to microvascular endothelial cells^44^. In addition, extracellular ANXA2 located on the cell surface also mediates plasminogen activation of endothelial cells^6, 8^, TLR4 signal-dependent macrophage activation^45^ and efferocytosis^16^. However, the mechanism of ANXA2 trafficking from cytosol to extracellular matrix is unclear. ANXA2, lacking a classical signal peptide, is suggested to be released to the extracellular matrix through an unconventional secretory pathway. It has been reported that ANXA2 is released to the extracellular matrix by exosomes in IFN-γ- and Ca^2+^-stimulated cells^16, 46^. In the present study, we observed that inhibition of autophagy by treatment with 3-MA or by *ATG5* knockdown significantly blocked exosomal secretion of ANXA2 after IFN-γ stimulation. We thus provide evidence that autophagy plays a role in the IFN-γ-stimulated ANXA2 secretory pathway.

Based on the results showing colocalization of ANXA2 with autophagosomes when stimulated by IFN-γ, we suggest that cytosolic ANXA2 protein is incorporated into autophagosomes after IFN-γ stimulation. Regarding how ANXA2 protein is incorporated into autophagosomes, recent report showed that ANXA2 promotes phagophore assembly in dendritic cells^47^. The selective autophagy occurs during normal growth conditions, which is different from the non-selective autophagy induced by stress^48^. During selective autophagy, ubiquitinated proteins are recognized by specific receptors (ex. p62 and NBR1) and targeted to the autophagosome via their interaction with LC3 protein conjugated on the lipid membrane of the autophagosome^48^. It has been suggested that polyubiquitinated ANXA2 is not degraded but has a function as actin-binding protein^49^. It remains to be further investigated whether ubiquitination of ANXA2 is required for ANXA2 incorporation into autophagosomes after IFN-γ treatment.

Based on our previous report^16^ and the findings in this study, exosomal release is an important pathway to export cytosolic ANXA2 to the extracellular matrix. Exosomes are generated from the fusion of intracellular MVBs with the plasma membrane and then release their accumulated small vesicles with a size of 40–100 nm into the extracellular matrix^37, 50^. Although MVBs serve as distinctive late endosomes, increasing evidence suggests that functional MVBs are closely related to the autophagic pathway. In fact, morphometric studies have shown that maturation of autophagy is a multistep process in mammalian cells, including the fusion of autophagosomes first with MVBs and then with lysosomes^38^. Furthermore, autophagy induction promotes the fusion of MVBs with autophagosomes, which is required for the maturation of autophagy in starvation-stimulated K562 cells^33^. In this study, overexpression of *RAB11S25N* mutant protein blocks the fusion of autophagosomes with MVBs, which is in agreement with a previous report^33^. The entry of ANXA2 into autophagosomes was not affected after IFN-γ stimulation upon overexpression of a *RAB11S25N* mutant. However, the colocalization of ANXA2 with amphisomes was significantly decreased. Our study shows that IFN-γ stimulation induces the interaction of autophagosomes with MVBs, which is important for further relocation of ANXA2 to the amphisomes.

The amphisome is generally characterized as a pre-lysosomal hybrid organelle resulting from the fusion of the MVB and the autophagosome, and has been known to fuse with the lysosome for protein degradation^38^. Here, we observe that the amphisome is also involved in ANXA2 secretion through an unconventional pathway in human lung epithelial cells. Extracellular secretion of ANXA2 by exosomal vesicles is decreased when the *RAB11S25N* mutant protein is overexpressed to block the formation of amphisomes. A recent study on reticulocytes has shown that the autophagosomes can combine with the endosomes to form large hybrid compartments which then fuse with the plasma membrane to release their contents by exocytosis^51^. Our observations show that amphisome formation is required for the extracellular secretion of ANXA2 via the exosomal pathway.

The question of how ANXA2 is relocated from the lumen of the autophagosome to the surface of the exosome remains to be resolved. We propose that cytosolic ANXA2 incorporated into the lumen of the autophagosome can be released in the amphisome after fusion of the autophagosome with the MVB. It is thought that the inner membrane of the autophagosome may be unstable and broken in the amphisome because the amphisome is more acidic (pH 5.7) than the autophagosome^28^. A further question remains as to whether ANXA2-containing exosomes are released through direct fusion of the amphisome with the plasma membrane or through a lysosome-dependent pathway. It was previously demonstrated that α-synuclein is secreted to the extracellular matrix via the direct fusion of the amphisome with the plasma membrane while p25α impairs the fusion of the amphisome with the lysosome in nerve cells^52^. On the other hand, the inhibition of autophagic flux by blocking fusion with the lysosome reduces autophagy-mediated IL-1β extracellular secretion in macrophages^25^. VAMP7 has been demonstrated to mediate the fusion of the amphisome with the lysosome. In our study, we observed that silencing *VAMP7* failed to block IFN-γ-stimulated secretion of ANXA2-containing exosomes. We suggest that the fusion of the amphisome with the plasma membrane to release ANXA2-containing exosomes is a predominant route for ANXA2 extracellular secretion by IFN-γ-stimulated lung epithelial cells. However, ANXA2 levels in exosomes are slightly less in *VAMP7*-silenced cells than control cells (Fig. 4c, *shVAMP7* vs. *shLuc*). We speculate that amphisome fusion with the lysosome to complete the autophagic flux may still be a minor route to secrete ANXA2-containing exosomes. This needs to be further confirmed.

Fusion of the amphisome with the plasma membrane is the final step of ANXA2 secretion. The mechanism of amphisome-mediated secretion is unclear. The secretion mediated by MVB, one of the amphisome predecessors, is regulated by RAB27A and RAB27B. The size of MVBs is increased in *RAB27A*-silenced cells, while MVBs are redistributed towards the perinuclear region in *RAB27B*-silenced cells^32^. Furthermore, RAB27A and granuphilin have been shown to regulate functional granule docking to the plasma membrane^53^. These reports suggest an important role of RAB27A in the fusion event between intracellular vesicle and plasma membrane. Our data show that silencing of *RAB27A*, but not *RAB27B*, inhibits ANXA2 secretion in cells. The role of RAB27B in regulating amphisome distribution but not fusion to the plasma membrane may be an explanation. RAB8A is involved in secretory autophagy^34^. However, the mechanism is not fully clear. We found that amphisome formation was still induced in *RAB8A* knockdown cells; however, ANXA2 release was inhibited. These results indicate that RAB8A regulates exophagy of ANXA2 in the fusion of the amphisome with the plasma membrane.

The release of exosomes into the extracellular space affords communication between cells by several mechanisms^54^. The exosomal membrane proteins can interact with target cell receptors. Exosomes can also fuse with the recipient cell membrane and release their contents. After fusion of exosomes with the recipient cell, the exosomal membrane proteins are incorporated into the surface membrane of the recipient cell. In this study, we found that the IFN-γ-enhanced surface expression of ANXA2 was reduced in *ATG5*, *RAB8A* and *RAB27A* knockdown cells (Supplementary Fig. 5), which correlated with the reduction of ANXA2-mediated efferocytosis (Fig. 6). These results indicate that the inhibition of exophagy results in the reduction of surface expression of ANXA2, which then leads to the inhibition of ANXA2-mediated efferocytosis.

Although we highlight the importance of exosomal secretion in autophagy-mediated ANXA2 exocytosis, other routes that may also be involved in ANXA2 exocytosis cannot be excluded. It is still unknown and it is worthy of further investigation whether the autophagosome directly fuses with the plasma membrane for ANXA2 exocytosis. Nevertheless, our study clarifies the exophagy route in ANXA2 exosomal secretion.

## Methods

### Antibodies

For western blotting, immunoprecipitation and immunofluorescence, the following antibodies were used: anti-ANXA2 (Abcam, ab54771, for confocal microscopy; BD, 610069, for western blotting), anti-CD63 (Abcam, ab18235), anti-Tsg101 (Abcam, ab83), mouse IgG (BD, 554121), anti-ATG5 (Cell Signaling Technology, 2630), anti-calnexin (Cell Signaling Technology, 2433), anti-LAMP1 (Cell Signaling Technology, 9091), anti-RAB7 (Cell Signaling Technology, 9367), anti-LC3 (MBL, PM036), anti-RAB8A (Proteintech, 55296-1-AP), anti-RAB27A (Proteintech, 17817-1-AP), anti-RAB27B (Proteintech, 13412-1-AP), anti-VAMP7 (R&D Systems, MAB6117), anti-cathepsin S (Santa Cruz, sc-74429), anti-SQSTM1/p62 (Santa Cruz, sc-28359), and anti-α-tubulin (Santa Cruz, sc-5286). For secondary staining in western blotting, the following antibodies were used: HRP-linked anti-mouse IgG (Cell Signaling Technology, 7076) and HRP-linked anti-rabbit IgG (Cell Signaling Technology, 7074). For secondary staining in immunofluorescence, the following antibodies were used: Alexa Fluor 488 goat anti-rabbit IgG (Life Technologies, A11034), Alexa Fluor 594 donkey anti-mouse IgG (Life Technologies, A21203), Alexa Fluor 594 donkey anti-rabbit IgG (Life Technologies, A21207), Alexa Fluor 647 donkey anti-mouse IgG (Life Technologies, A31571), and Alexa Fluor 647 donkey anti-rabbit IgG (Life Technologies, A31573).

### Cell cultures

Human lung adenocarcinoma cell line A549 (ATCC CCL-185) and human embryonic kidney 293 T cells (ATCC CRL-3216) were cultured in DMEM (GIBCO, 12800-017) and Jurkat T cells (ATCC TIB-152) were cultured in RPMI-1640 (Hyclone, SH30027.02). All media contained penicillin, streptomycin, amphotericin (Biological Industries, 03-033-1B) and 10% fetal bovine serum (FBS; Biological Industries, 04-001-1A). Cells were cultured at 37 °C in a humidified atmosphere of 5% CO_2_. 293T and Jurkat T cells were used for lentivirus production and phagocytosis assay, respectively.

### Cell treatment

Cells were cultured with or without 500 U/ml IFN-γ (Cell Guidance Systems, GFH77) in growth medium containing 10% FBS for different time periods. 3-MA-inhibited autophagy induction and lysosomal degradation can be prevented through the use of protease inhibitor such as bafilomycin A1^55^. Inhibitors including 3-MA (Calbiochem, 189490) and bafilomycin A1 (Millipore, 196000) were pre-incubated with cells for 1 h before IFN-γ treatment. Inhibitors were maintained in the culture during the incubation period.

### Exosome isolation

Because FBS contains endogenous exosomes, the culture medium containing 10% FBS was centrifuged overnight at 110,000 *g* to remove endogenous exosomes. After IFN-γ stimulation, exosomes in the cell culture medium were isolated. Briefly, the culture medium was collected and centrifuged at 300 *g* for 10 min to remove suspended cells. The supernatant was then centrifuged at 2,000 *g* for 10 min to remove the dead cells. After centrifugation at 10,000 *g* for 30 min to remove cell debris, the exosomes from the supernatant were centrifuged at 110,000 *g* for 70 min. The exosomal pellet was washed once in phosphate-buffered saline (PBS) and then resuspended in 200 μl PBS.

### Plasmid transfection

The *pcDNA*-*RAB11wt* and *pcDNA*-*RAB11S25N* were kindly provided by Dr. Mindong Ren (Department of Cell Biology, New York University, USA). Five μg plasmids were transfected into 5 × 10^6^ A549 cells by microperator (Invitrogen) using the condition: 1500 V, 30 ms, and 1 pulse time. Cells were incubated for 24 h and then treated with IFN-γ for subsequent experiments. *GFP*-*LC3* vector was kindly provided by Dr. Tamotsu Yoshimori (Laboratory of Intracellular Membrane Dynamics, Graduate School of Frontier Biosciences, Osaka University, Japan) and *CD63*-*GFP* was originally from Dr. Paul Luzio (Cambridge University, United Kingdom) and obtained from Addgene (plasmid # 62964). One μg of *CD63*-*GFP* or *GFP*-*LC3* vector and 2 μl of TurboFect (Thermo, R0531) were diluted in 400 μl of serum-free DMEM and incubated for 20 min at room temperature. Mixtures were added to A549 cells and incubated for 48 h at 37 °C.

### Generation of lentivirus shRNA and infection of A549 cells

293T cells were transfected with specific gene-knockdown vectors and the packaging vectors (pMD.G and pCMV-ΔR8.91) by using the transfection reagent GeneJammer (Agilent Technologies, 204130-21). The culture medium was changed after 24 h and collected after 48 h. The supernatant was filtered through a 0.22 μm filter (PALL, PN4602) to remove floating cells. The supernatant was centrifuged at 20,000 *g* for 4 h. The viral pellet was suspended in 200 μl culture medium and was used to infect A549 cells. For stable silenced selection, cells were cultured in the medium containing 2.5 μg/ml puromycin (Sigma, P8833). The specific gene-knockdown vectors were obtained from National RNAi Core Facility, Taiwan, with the following sequences: *Luciferase*, 5′-GCGCCATTCTATCCGCTGGAA-3′; *ATG5*, 5′-CGGGATGCTTTGAACATTGAA-3′; *VAMP7*, 5′-TCTTATGAGCTATCTACTAAA-3′; *RAB7*, 5′-GGCTAGTCACAATGCAGATAT-3′; *RAB8A*, 5′-CGGAACTGGATTCGCAACATT-3′; *RAB27A*, 5′-CGGATCAGTTAAGTGAAGAAA-3′; *RAB27B*, 5′-CATCATCATGGATACTCAATT-3′.

### Western blotting

Cells were lysed in 100 μl lysis buffer (20 mM Tris-HCl, pH 7.5, 1% Triton X-100, 150 mM NaCl, 5 mM EDTA, 100 mM sodium pyrophosphate, 1 mM β-glycerolphosphate, 10 mM sodium orthovanadate, and 2 mM PMSF) with protease inhibitor cocktail (MDBio, 2043). Sodium pyrophosphate and β-glycerophosphate were used to be the false substrates for phosphatase in general. For immunoblotting, the total protein content was determined using the Bradford assay. Total cell lysates were mixed with 1/4 volume of sample buffer (250 mM Tris-HCl, 500 mM DTT, 10% SDS, 0.1% bromophenol blue, and 50% glycerol) for 5 min at 95 °C and separated by SDS-PAGE. Proteins were transferred to polyvinylidene difluoride membranes (Millipore, IPVH00010). After blocking with 5% nonfat milk in PBS-T (0.05% Tween-20), blots were washed with PBS-T for three times, then probed with primary antibodies at 4 °C overnight. After PBS-T washing, blots were stained with HRP-conjugated secondary antibodies in 1/5000 dilution at room temperature for 1 h. Blots were developed using Western lightning chemiluminescence reagent (Millipore, WBKLS0500; PerkinElmer, NEL105001EA).

### Immunofluorescence

Cells were fixed with 1% formaldehyde/PBS and then permeabilized with permeabilization buffer (1% bovine serum albumin, 0.05% NaN_3_, and 0.1% saponin) for 10 min at room temperature. Cells were stained with primary antibodies at 4 °C for 24 h followed by secondary antibodies for 1 h at room temperature. The nuclei were subsequently stained with 1 μg/ml DAPI at room temperature for 10 min. Cells were mounted by Fluoromount-G (Electron Microscopy Sciences, 17984-25) and analyzed by confocal microscopy (Olympus, FV1000). Laser wavelengths of 405 nm, 473 nm 559 nm and 635 nm were used and the images were obtained from 0.2 μm sections.

### Phagocytosis assay

A549 cells were treated with or without IFN-γ (500 U/ml) for 48 h and Jurkat T cells were cultured in serum-free RPMI-1640 with 50 μM etoposide (Sigma, E1383) for 24 h. After etoposide treatment, Jurkat T cells were collected and co-cultured with A549 cells for 2 h. After 2 h incubation, non-ingested apoptotic cells were removed by vigorous washing with 0.02 M EDTA/PBS. Cells were fixed with 1% formaldehyde/PBS and then permeabilized with permeabilization buffer for 10 min at room temperature. After permeabilization, cells were stained with DAPI and rhodamine-phalloidin (Cytoskeleton, Inc., PHDR1) for nucleus and F-actin. Cells were mounted and analyzed by confocal microscopy.

### Statistical analysis

Pearson’s colocalization coefficients were measured by using ImageJ software and quantification was performed by using GraphPad Prism software. Results from three independent experiments were shown as the mean ± SD. Data obtained from cells with two different treatments were compared and analyzed by two-tailed unpaired Student’s *t* test. Data obtained from cells with three or more different treatments were compared and analyzed by Tukey’s one-way ANOVA. The *P* value of less than 0.05 was considered to be significant.

## Electronic supplementary material

Supplementary information
