# Supplementary material for: Exophagy of annexin A2 via RAB11, RAB8A and RAB27A in IFN-γ-stimulated lung epithelial cells
Source: Sci Rep. 2017 Jul 18;7:5676. doi: 10.1038/s41598-017-06076-4 (PMC5516008; doi:10.1038/s41598-017-06076-4)
Supplement: Supplementary file 1 — Supplementary information [file 41598_2017_6076_MOESM1_ESM.pdf]

# Supplementary Information

## Exophagy of annexin A2 via RAB11, RAB8A and RAB27A in IFN-γ-stimulated lung epithelial cells

Ying-Da Chen, Yi-Ting Fang, Yi-Lin Cheng, Chiou-Feng Lin, Li-Jin Hsu, Shu-Ying Wang, Robert Anderson, Chih-Peng Chang* & Yee-Shin Lin*

* Correspondence and requests for materials should be addressed to C.P.C. (email: cpchang@mail.ncku.edu.tw) or to Y.S.L. (email: yslin1@mail.ncku.edu.tw)

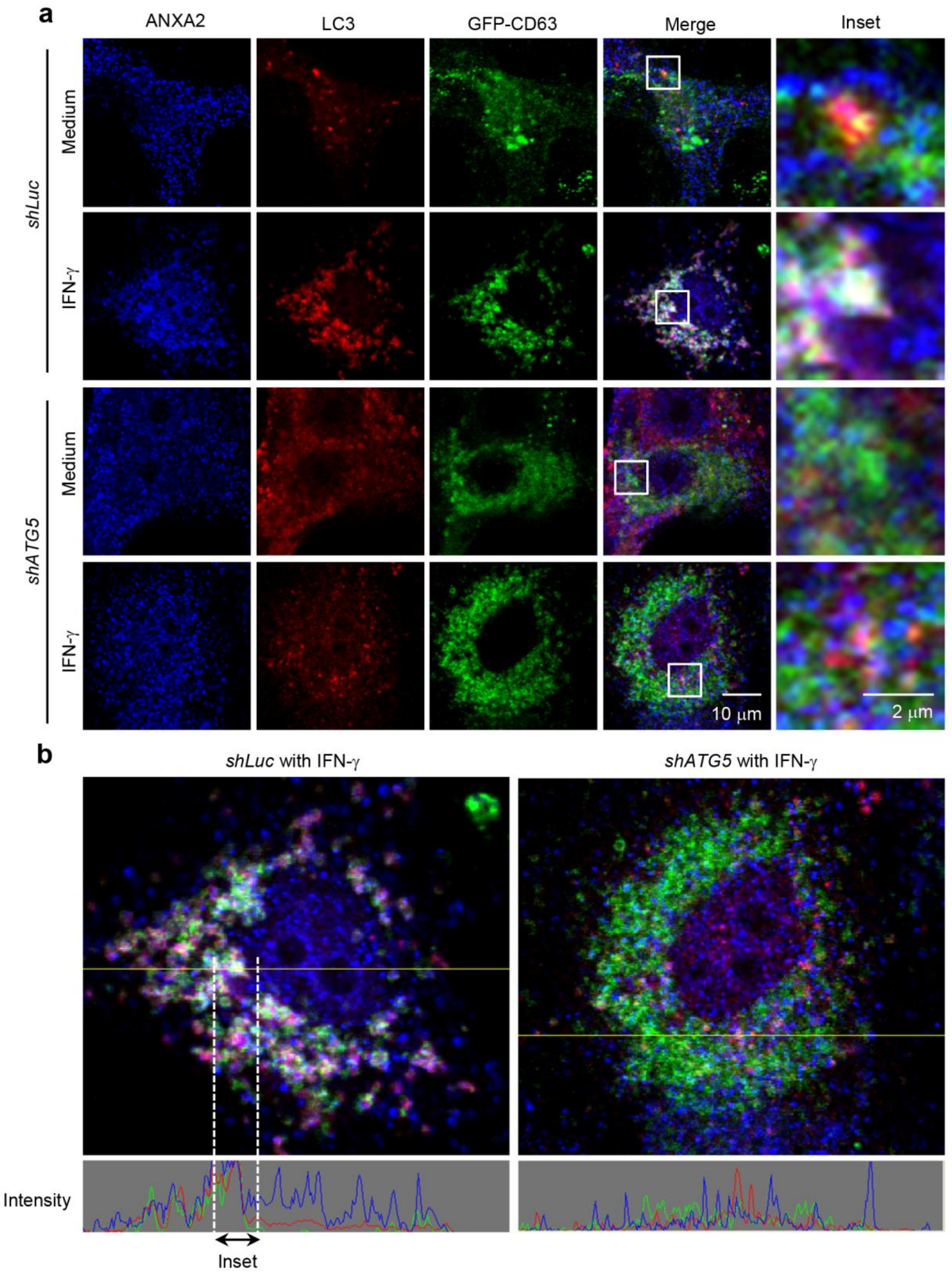


**Supplementary Figure 1. IFN-γ-induced ANXA2-containing amphisomes are reduced in *ATG5* knockdown cells.** (**a**) Cells with stable *ATG5* knockdown and control knockdown were transfected with CD63-GFP plasmid and then treated with or without 500 U/ml IFN-γ for 24 h. Cells were then fixed, permeabilized, and stained

for ANXA2 (blue) and LC3 (red). The colocalization of ANXA2, LC3 and CD63-GFP was observed by confocal microscopy. Scale bar: 10 μm (2 μm in insets). (**b**) Line tracing analysis of fluorescence signal from image in (**a**) of *ATG5* knockdown and control knockdown cells after IFN-γ stimulation is shown.

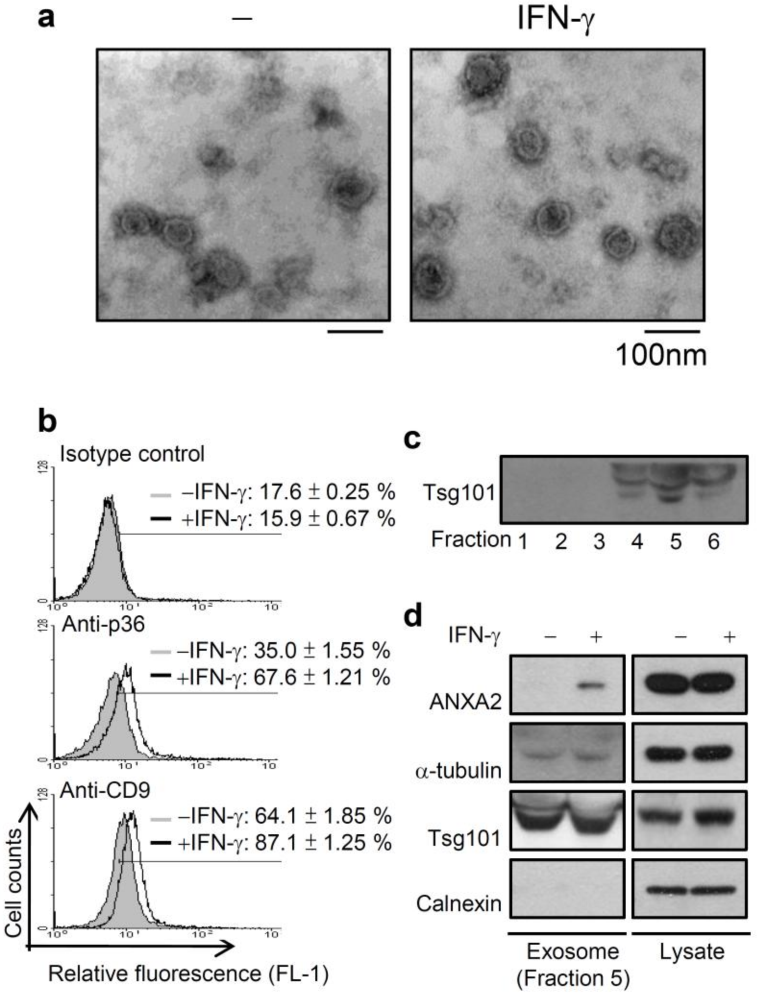


**Supplementary Figure 2. IFN-γ enhances exosomal secretion of ANXA2.** (**a**) A549 cells were treated with or without 500 U/ml IFN-γ for 48 h. The exosome pellets were collected and observed by transmission electron microscopy. (**b**) The exosome pellets were collected after serial centrifugation and the exosome marker, CD9, and surface ANXA2 of exosomes were detected by flow cytometry. (**c**) The exosome pellets in different fractions of sucrose gradient were collected and Tsg101 (exosome marker) was detected by western blotting. Fractions 1 to 6 were collected from the bottom to the top. (**d**) A549 cells were treated with or without 500 U/ml IFN-γ for 48 h. The

exosome pellets in the sucrose gradient fraction 5 were collected and ANXA2, α-tubulin, Tsg101 and calnexin from exosome pellets and total cell lysates were detected by western blotting.

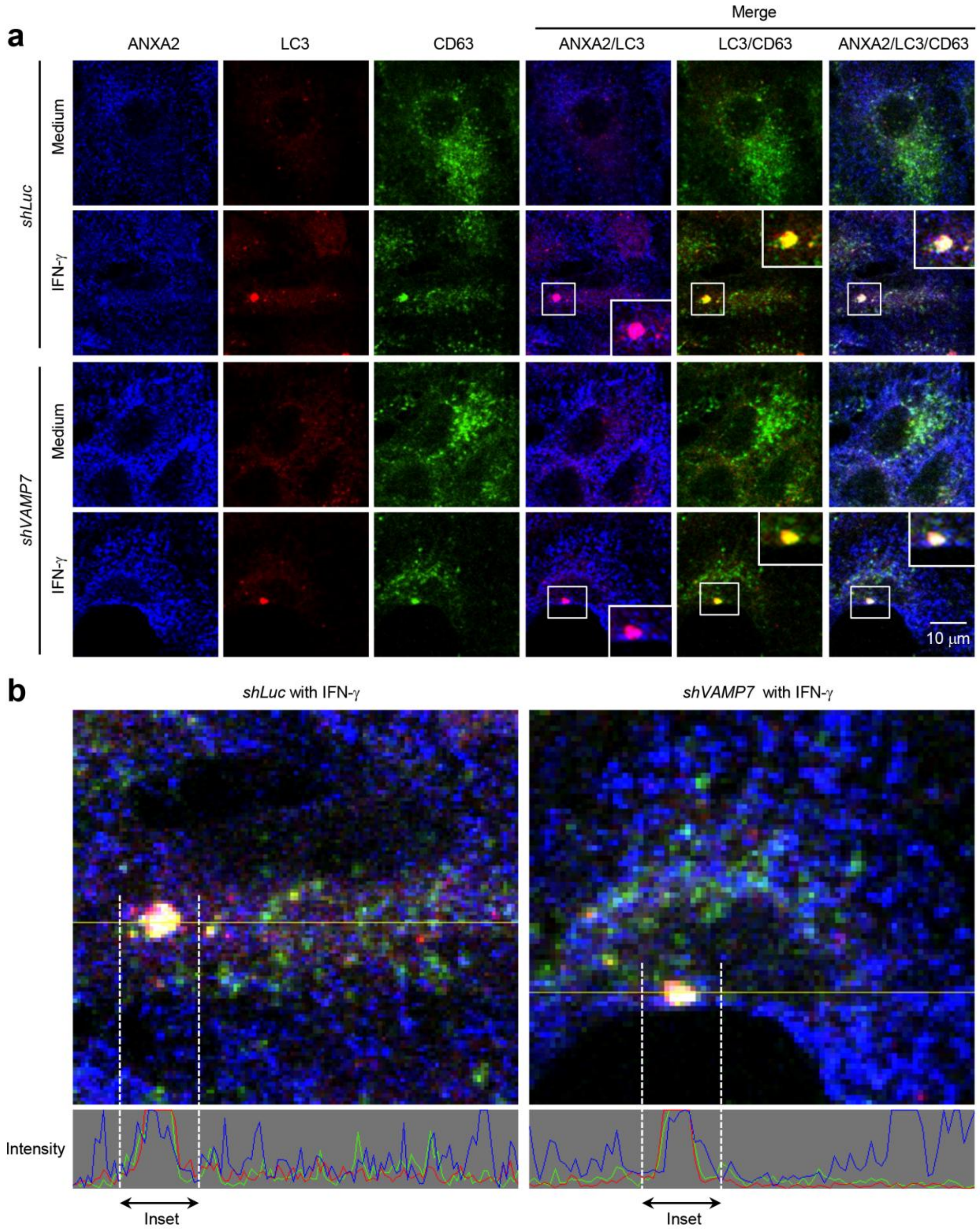


**Supplementary Figure 3. IFN-γ-induced amphisome formation is not affected by knockdown of *VAMP7*.** (**a**) Cells with stable *VAMP7* knockdown and control knockdown were treated with or without 500 U/ml IFN-γ for 24 h. Cells were then fixed, permeabilized, and stained for ANXA2 (blue), LC3 (red) and CD63 (green). The colocalization of ANXA2, LC3 and CD63 was observed by confocal microscopy.

Scale bar: 10 μm. (**b**) Line tracing analysis of fluorescence signal from image in (**a**) of *VAMP7* knockdown and control knockdown cells after IFN-γ stimulation is shown.

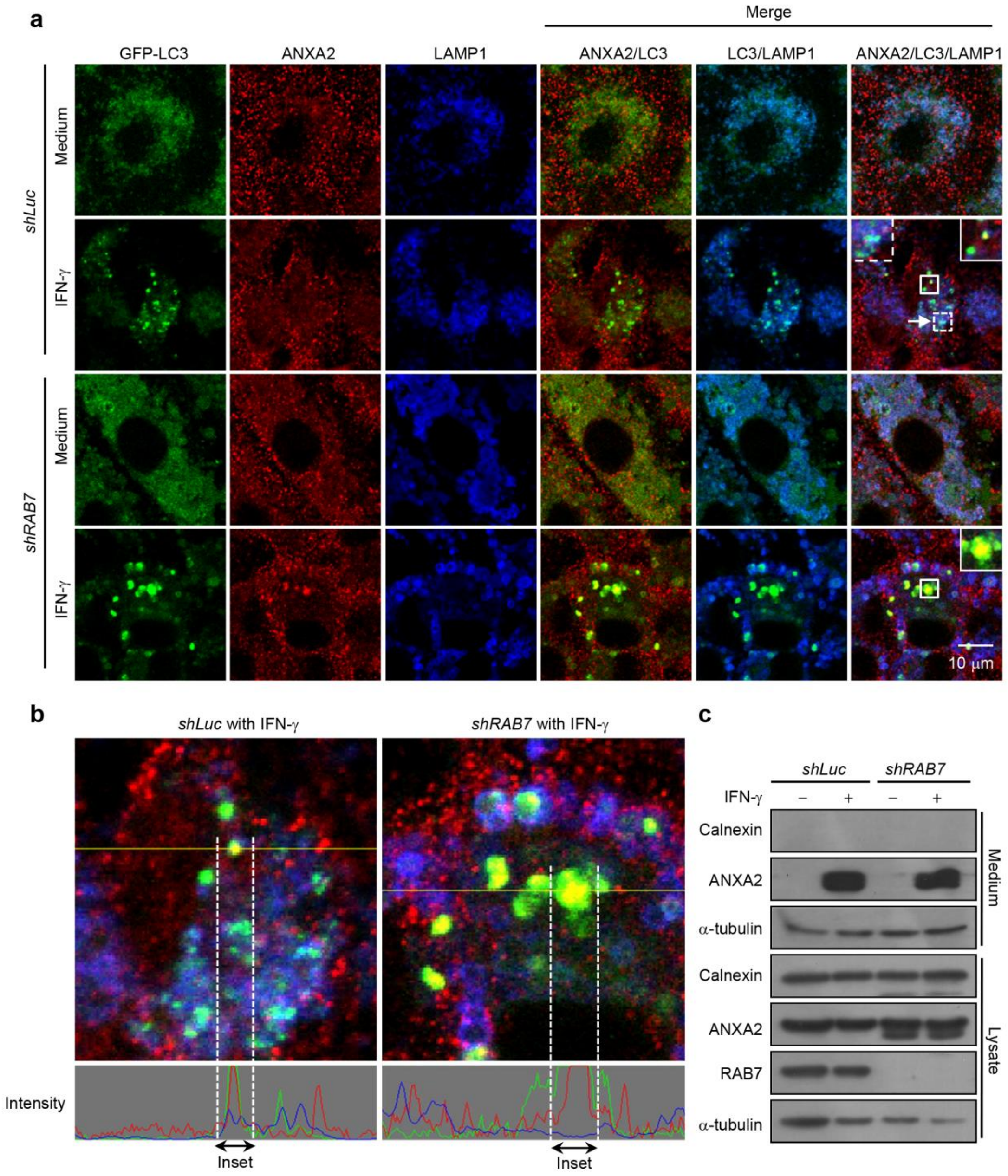


**Supplementary Figure 4. Amphisome/lysosome fusion is not essential for IFN-γ-induced ANXA2 release.** (**a**) Cells with *RAB7* knockdown and control knockdown were transfected with a *GFP-LC3* plasmid and treated with or without 500 U/ml IFN-γ for 24 h. Cells were then fixed, permeabilized, and stained for ANXA2 (red) and LAMP1 (blue). The colocalization of ANXA2, GFP-LC3 and LAMP1 was observed by confocal microscopy. Scale bar: 10 μm. The arrow and

dotted inset indicate an autolysosome. (**b**) Line tracing analysis of fluorescence signal from image in (**a**) of *RAB7* knockdown and control knockdown cells after IFN-γ stimulation is shown. (**c**) Control and *RAB7*-silenced cells were treated with or without 500 U/ml IFN-γ for 48 h. Calnexin, ANXA2 and α-tubulin from cultured supernatant and total cell lysate were analyzed by western blotting.

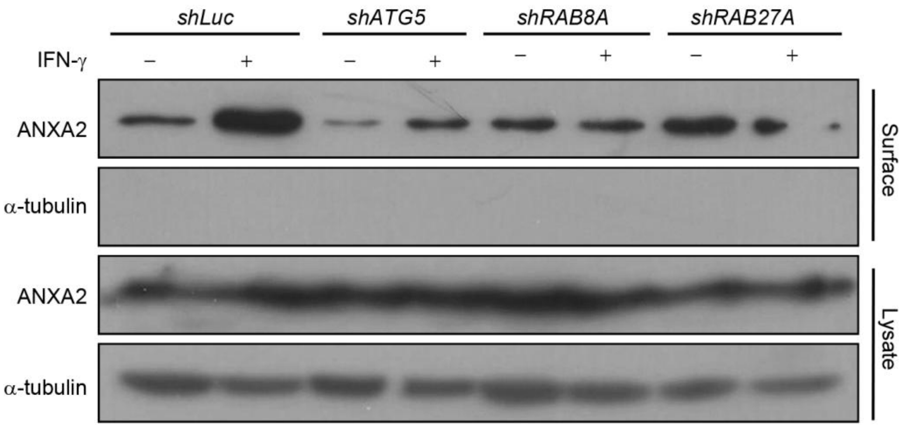


**Supplementary Figure 5. IFN-γ-induced ANXA2 surface expression is inhibited in *ATG5*, *RAB8A* and *RAB27A* knockdown cells.** *ATG5*, *RAB8A*, *RAB27A* and control knockdown cells were treated with or without 500 U/ml IFN-γ for 48 h. Cells were eluted with 20 mM EGTA for 30 min at 4℃ to remove surface ANXA2. Eluted ANXA2 and α-tubulin from surface and total cell lysate were analyzed by western blotting.

**Supplementary methods**

**Transmission electron microscopy.** A549 cells were treated with or without 500 U/ml IFN-γ for 48 h. The exosome pellets were collected from medium after serial centrifugation and fixed with 2.5% glutaraldehyde in 0.1 M cacodylate buffer containing 5% sucrose. The exosome pellets were fixed in 1% osmium tetroxide and dehydrated with ethanol. After dehydration, cells were embedded with Embed-812. Ultrathin sections were stained with uranyl acetate and lead citrate and imaged on transmission electron microscope.

**Flow cytometry for exosomes.** A549 cells were treated with or without 500 U/ml IFN-γ for 48 h and the exosome pellets were collected from medium after serial centrifugation. Exosomes were incubated with 4 μm diameter aldehyde sulfate latex beads (ThermoFisher, A37304) for 15 min at room temperature. After overnight incubation at 4℃, the reaction was stopped by the addition of 100 mM glycine for 30 min. Exosome-coated beads were washed by PBS and stained for control IgG (Santa Cruz, sc-2027), ANXA2 (Abcam, ab54771) and CD9 (Abcam, ab65230), respectively, at room temperature for 1 h. Following PBS washing, the antibody-exosome-coated beads were stained with Alexa 488-labeled secondary antibody (Life Technologies, A11034) and detected by flow cytometer (BD, FACSCalibur).

**Cell surface elution.** Surface ANXA2 was eluted as described previously[16]. Briefly, *ATG5*, *RAB8A*, *RAB27A* and control knockdown cells were treated with or without 500 U/ml IFN-γ for 48 h. After treatment, cells were washed three times with ice-cold Hepes-buffered saline (HBS) and eluted with HBS buffer containing 20 mM EGTA and protease inhibitors at 4℃ for 30 min. The eluates and total cell lysates were collected, respectively, and detected by western blotting.

**Sucrose gradient fractionation.** The sucrose gradient was prepared as 1.5 ml each of 60%, 50%, 40%, 30%, 20%, and 5% sucrose. Using differential centrifugation, 2 ml of exosome isolates were loaded onto the top of the gradient, followed by immediate centrifugation at 280,000 *g* for 18 h. After centrifugation, 1.5 ml fractions were collected from the bottom of the gradient designated fraction number 1 (bottom) through 6 (top). The fractions were then diluted in 10 ml PBS and concentrated with centrifugation at 280,000 *g* for 1 h. The pellets were resuspended in 1X SDS-PAGE loading buffer and boiled. Then, 30 μl of each sample was separated on SDS-PAGE followed by western blotting.
